# Human Borna disease virus 1 (BoDV-1) encephalitis cases in the north and east of Germany

**DOI:** 10.1080/22221751.2021.2007737

**Published:** 2021-12-21

**Authors:** Christina Frank, Jonathan Wickel, Dirk Brämer, Jakob Matschke, Richard Ibe, Caroline Gazivoda, Albrecht Günther, Christian Hartmann, Kordt Rehn, Daniel Cadar, Thomas E. Mayer, Kirsten Pörtner, Hendrik Wilking, Jonas Schmidt-Chanasit, Dennis Tappe

**Affiliations:** a Department for Infectious Disease Epidemiology, Robert Koch Institute, Berlin, Germany; b Department of Neurology, Section of Translational Neuroimmunology, Jena University Hospital, Jena, Germany; c Hans Berger Department of Neurology, University Hospital Jena, Jena, Germany; d Institute for Neuropathology, University Hospital Hamburg-Eppendorf, Hamburg, Germany; e Department of Neurology, University Hospital Halle/Saale, Halle/Saale, Germany; f Department of Neuropathology, Institute of Pathology, Hannover Medical School (MHH), Hannover, Germany; g Independent researcher, Bad Pyrmont, Germany; h Bernhard Nocht Institute for Tropical Medicine, Hamburg, Germany; i Department of Neuroradiology, University Hospital Jena, Jena, Germany

**Keywords:** Borna disease, Borna disease virus 1, bornavirus, surveillance, epidemiology

## Abstract

In 2021, three encephalitis cases due to the Borna disease virus 1 (BoDV-1) were diagnosed in the north and east of Germany. The patients were from the states of Thuringia, Saxony-Anhalt, and Lower Saxony. All were residents of known endemic areas for animal Borna disease but without prior diagnosed human cases. Except for one recently detected case in the state of Brandenburg, all >30 notified cases had occurred in, or were linked to, the southern state of Bavaria. Of the three detected cases described here, two infections were acute, while one infection was diagnosed retrospectively from archived brain autopsy tissue samples. One of the acute cases survived, but is permanently disabled. The cases were diagnosed by various techniques (serology, molecular assays, and immunohistology) following a validated testing scheme and adhering to a proposed case definition. Two cases were classified as confirmed BoDV-1 encephalitis, while one case was a probable infection with positive serology and typical brain magnetic resonance imaging, but without molecular confirmation. Of the three cases, one full virus genome sequence could be recovered. Our report highlights the need for awareness of a BoDV-1 etiology in cryptic encephalitis cases in all areas with known animal Borna disease endemicity in Europe, including virus-endemic regions in Austria, Liechtenstein, and Switzerland. BoDV-1 should be actively tested for in acute encephalitis cases with residence or rural exposure history in known Borna disease-endemic areas.

## Introduction

The Borna disease virus 1 (BoDV-1; species *Mammalian orthobornavirus* 1) is one of the two known zoonotic members of the *Bornaviridae* family. For a long time, BoDV-1 has been noted to cause animal Borna disease (BD), a non-purulent meningomyelo-encephalitis of mainly horses and sheep in endemic regions of Germany, Liechtenstein, Switzerland and Austria [1,2]. BoDV-1 is harboured by at least the insectivorous bicoloured white-toothed shrew (*Crocidura leucodon*) as a natural reservoir [1,3]. In 2018, the severe pathogenic potential of BoDV-1 for humans became apparent in a cluster of transplant-related BoDV-1 encephalitis cases in Germany with two fatalities and one significantly disabled survivor [4]. Simultaneously, one sporadic unrelated fatal case was detected [5]. Since then, nearly 40 sporadic cases, some acute and some retrospectively diagnosed, have been published in the literature [5–10] and/or were notified and transmitted to the Robert Koch Institute on the national level. All these cases were fatal, and, prior to the cases described here, all but one patient lived in BD endemic areas in the southern German state of Bavaria. North of Bavaria, previously only one confirmed BoDV-1 encephalitis case living in the state of Brandenburg had been retrospectively diagnosed in 2020 from archived autopsy material [11]. BoDV-1 is believed to have a high case-fatality rate and the virus may be responsible for a considerable proportion of fatal encephalitis cases of previously unknown origin [6,7]. To intensify surveillance, the direct detection of bornaviruses in human samples was made legally notifiable by the German Infection Protection Act (Infektionsschutzgesetz, IfSG) in 2020.

We here report three additional cases of human BoDV-1 encephalitis outside of Bavaria, in the north and east of Germany, including two cases diagnosed in the acute phase. The patients, one of whom survived, lived in BD endemic regions without previously recognized human infections. The two acute cases were detected by a recently validated diagnostic workflow following a nationwide awareness initiative for human bornavirus encephalitis, while a third case was detected retrospectively from a neuropathology archive. We provide clinical, histopathological, molecular and epidemiological data of these cases and present implications for surveillance of human bornavirus encephalitis.

## Materials and methods

### Case detection and clinical case definitions

A recently validated diagnostic workflow for the rapid *intra vitam* diagnosis of human bornavirus encephalitis [7] was employed for testing serum and cerebrospinal fluid (CSF) of patients with cryptic encephalitis that were sent to the Bernhard Nocht Institute in Hamburg, Germany. In addition, neuropathology depositories in the north and east of Germany were screened for archived brain tissues from lethal encephalitis cases with unknown etiology. Retrieved cases were analyzed by molecular tools and immunostaining for BoDV-1 infections. Ethical clearance was obtained from the local ethics board (Medical Board of Hamburg, no. PV5616).

A proposed graded case definition for possible, probable and confirmed BoDV-1 encephalitis [7] was used in parallel which is based on international consensus criteria for encephalitis [12].

### Serology

The serologic workflow consists of an indirect immunofluorescence assay (IFAT) with a persistently BoDV-1 infected cell line for screening, followed by a line blot assay for confirmation [7]. The line blot utilizes recombinant BoDV-1 phosphoprotein (P) antigen with a cut-off of 16 arbitrary units (AU). Sera and CSF samples of patients with confirmed BoDV-1 encephalitis served as positive controls for both the IFAT, demonstrating a specific intranuclear pattern, and for the line blot.

### Polymerase chain reaction, next-generation sequencing and viral phylogeny inferences

Quantitative reverse-transcription real time polymerase chain reaction (qRT-PCR) for BoDV-1 [4] was conducted from CSF of seropositive patients and from available formalin-fixed paraffin-embedded (FFPE) brain tissues. For complete virus genome reconstruction, a diagnostic sample underwent unbiased next-generation sequencing (NGS) using a NextSeq550 Illumina sequencing platform as described elsewhere [13].

Molecular relationships of BoDV-1 sequences were analyzed by constructing a phylogenetic tree using the maximum likelihood method in PhyML 3.0 (https://www.atgc-montpellier.fr/phyml/versions.php) with 1000 pseudo-replicates based on sequences from the nucleoprotein to the phosphoprotein gene (1824 nt, representing genome positions 54–1877 of BoDV-1 reference genome U04608). For the assessment of specific node support, subtree pruning and regrafting (SPR), branch-swapping and an approximate likelihood ratio test (aLRT) were performed. The Akaike information criterion was chosen as the model selection framework and the GTR + I + G as the best model.

### Histology, immunohistochemistry and in situ-hybridization

Available FFPE brain tissue was processed for routine histology with hematoxylin and eosin stains. Immunohistology for BoDV-1 P antigen was performed as described elsewhere [14], using rabbit polyclonal sera. For this purpose, rabbits were immunized with recombinantly expressed BoDV-1 P antigen [14]. Immunohistology for CD3, CD4, CD8, CD20 and CD68 was conducted as also described elsewhere [14], using commercially available antibodies.

*In situ*-hybridization for BoDV-1 RNA using the V-BoDV1-G probe (RNAScope, Advanced Cell Diagnostics/Bio-Techne, Abingdon, UK) was performed according to the manufacturer’s instructions.

## Results

Two acute cases (cases 1 and 2) with BoDV-1 encephalitis were detected, one of them fatal. In addition, one fatal case (case 3) was retrospectively diagnosed from a neuropathology archive.

**Figure 1.**
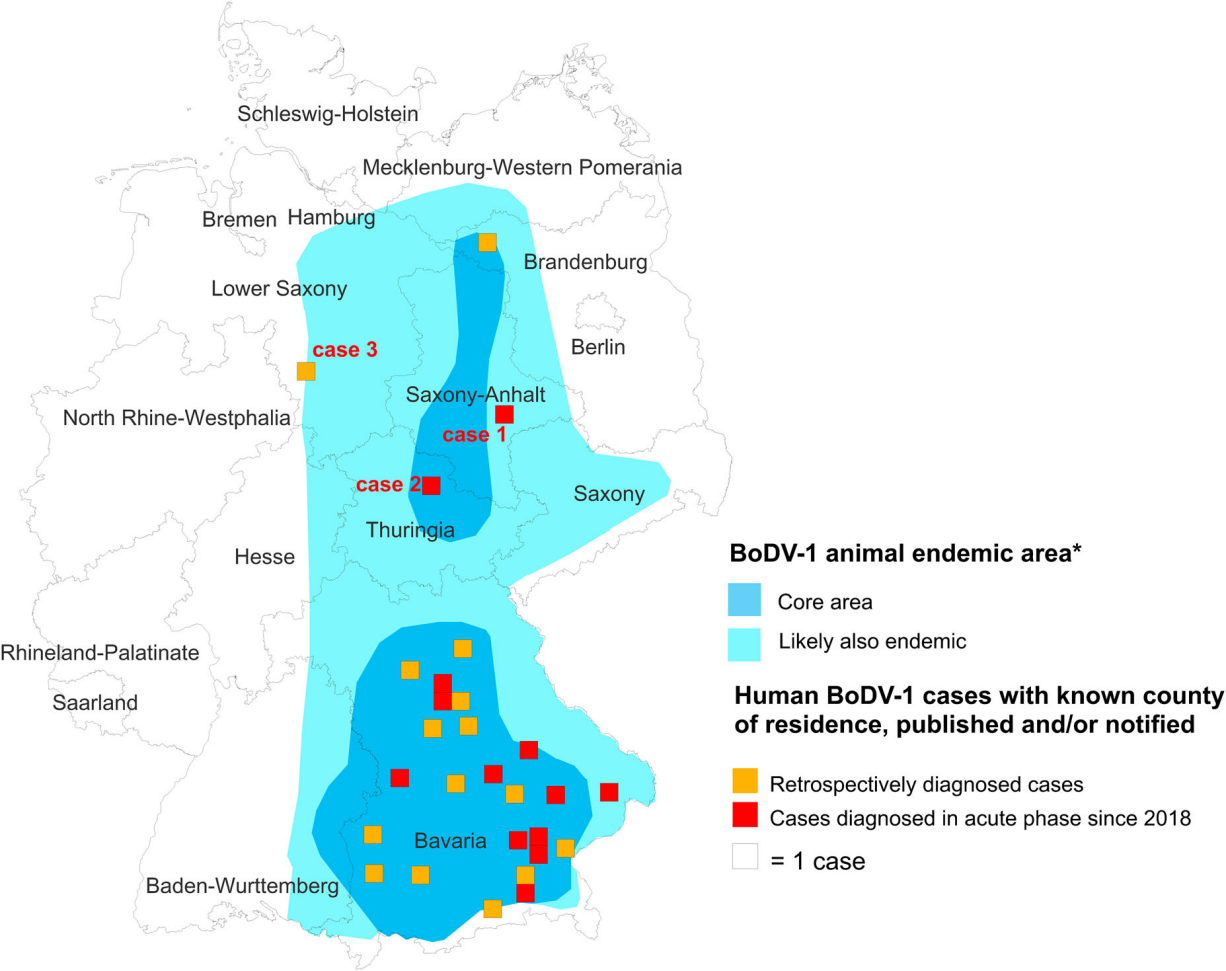
Geographical location of the human BoDV-1 encephalitis cases of this report and other cases with known county of residence (published/notified) in relation to the virus-endemic area. The human cases reported in this study are shown together with previously published BoDV-1 infections of humans (yellow and orange squares) in the map. The area known to be endemic for animal Borna disease or presence of BoDV-1 positive shrews is represented by the two shades of blue (*Source: https://www.rki.de/DE/Content/InfAZ/B/Bornavirus/Merkblatt.pdf?__blob=publicationFile).

**Figure 2.**
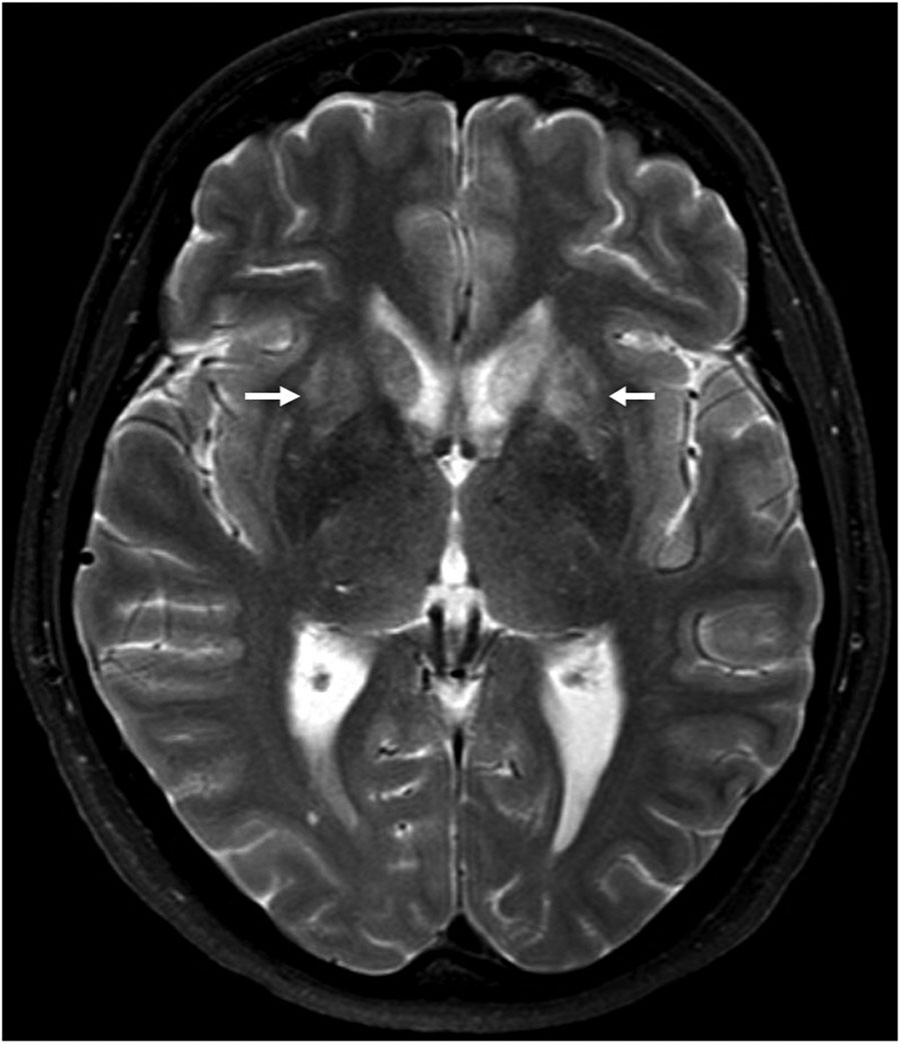
Cranial magnetic resonance imaging of case 1. The typical affection of the basal ganglia (arrows) as found in acute BoDV-1 encephalitis [10] is shown in this case of a probable BoDV-1 encephalitis according to the case definition criteria [7]. Transversal T2-weighted image.

**Case 1** was diagnosed in January 2021. The patient is a 58-year-old housewife from a rural region of Saxony-Anhalt (Figure 1) on the Elbe River. From late December 2020 she developed dysphasia, vigilance decline and epileptic seizures, followed by sopor and ocular bulbus divergence. Magnetic resonance imaging (MRI) of the brain showed diffuse edema and hyperintensities temporomesial, insular, in the basal ganglia, and posterior thalami (Figure 2). At the time of diagnosis, IFAT titre was 20,480 for serum and 1280 for CSF. Line blot results for antibodies against BoDV-1 P antigen were 34 AU for serum and 40 AU for CSF. qRT-PCR was negative from CSF. Diagnosis was made three weeks after onset of symptoms. As of November 2021, the patient is alive but is left severely disabled and lives in a nursing home. According to the proposed case definition criteria [7], this case is categorized as probable bornavirus encephalitis, as no molecular confirmation was possible yet.

**Figure 3.**
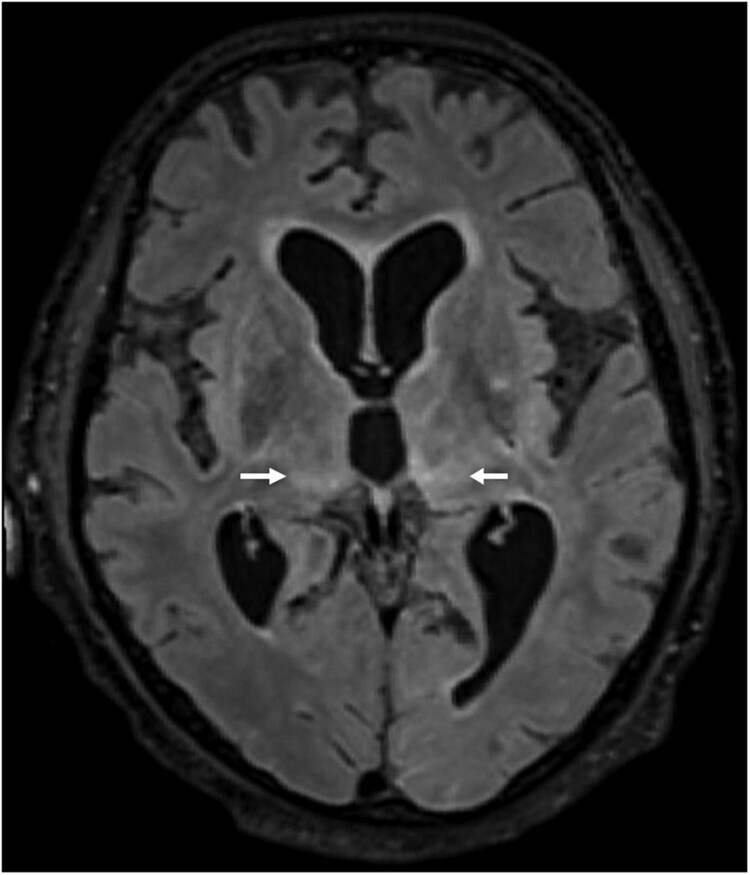
Cranial magnetic resonance imaging of case 2. In this case of a confirmed BoDV-1 encephalitis, widespread increase of signal intensity, especially of the thalami (arrows) is detected. Transversal T2-weighted fluid attenuated image (FLAIR).

**Figure 4.**
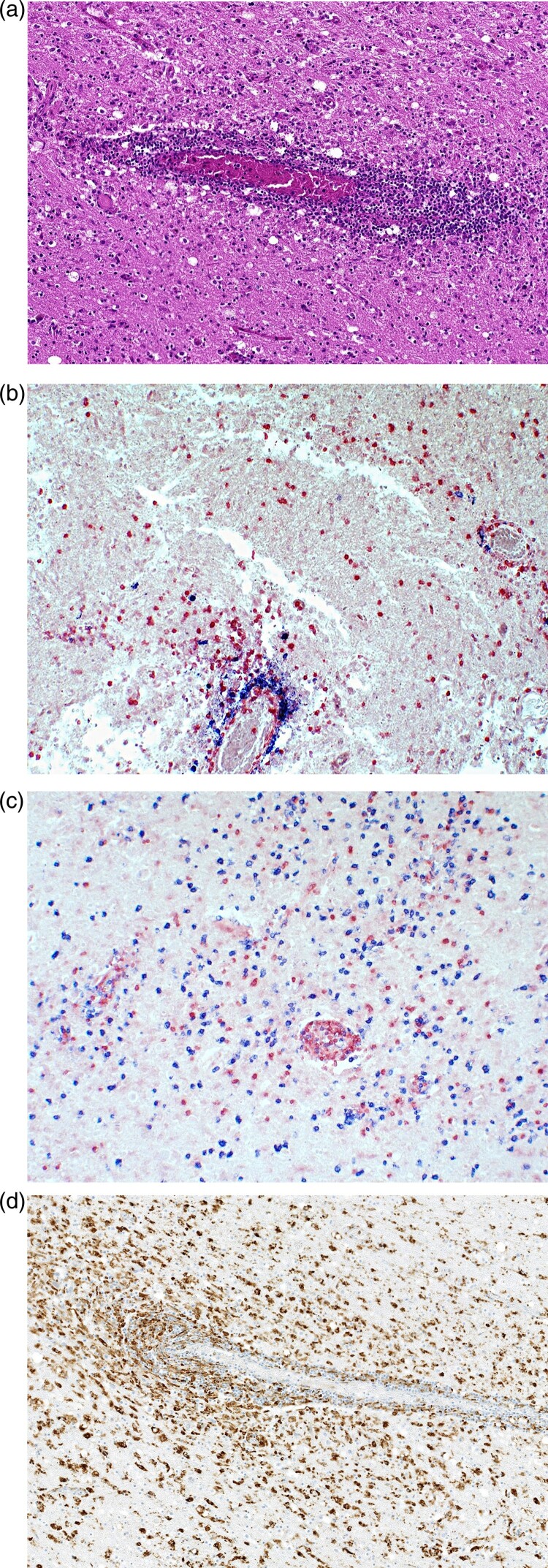
Histopathologic findings of BoDV-1 encephalitis (case 2). A, Severe inflammation as reflected by mononuclear cell infiltration and perivascular cuffing. Hematoxylin and eosin stain of the thalamus, original magnification ×50. B, Perivascular accumulation of B cells (CD20, blue) and T cells (CD3, red). While B cells demonstrated mainly perivascular cuffing, T cells were additionally infiltrating the brain parenchyma in high numbers. Immunoperoxidase and immunophosphatase stains of the hippocampus, original magnification ×100. C, Distribution of CD4+ (red) and CD8+ (blue) T lymphocytes in inflammatory infiltrates. Immunoperoxidase and immunophosphatase stains of the hippocampus, original magnification ×100. D, Microglia activation in inflamed regions of the brain. Immunoperoxidase stain for CD68 with hematoxylin counterstain of the thalamus, original magnification ×50.

**Figure 5.**
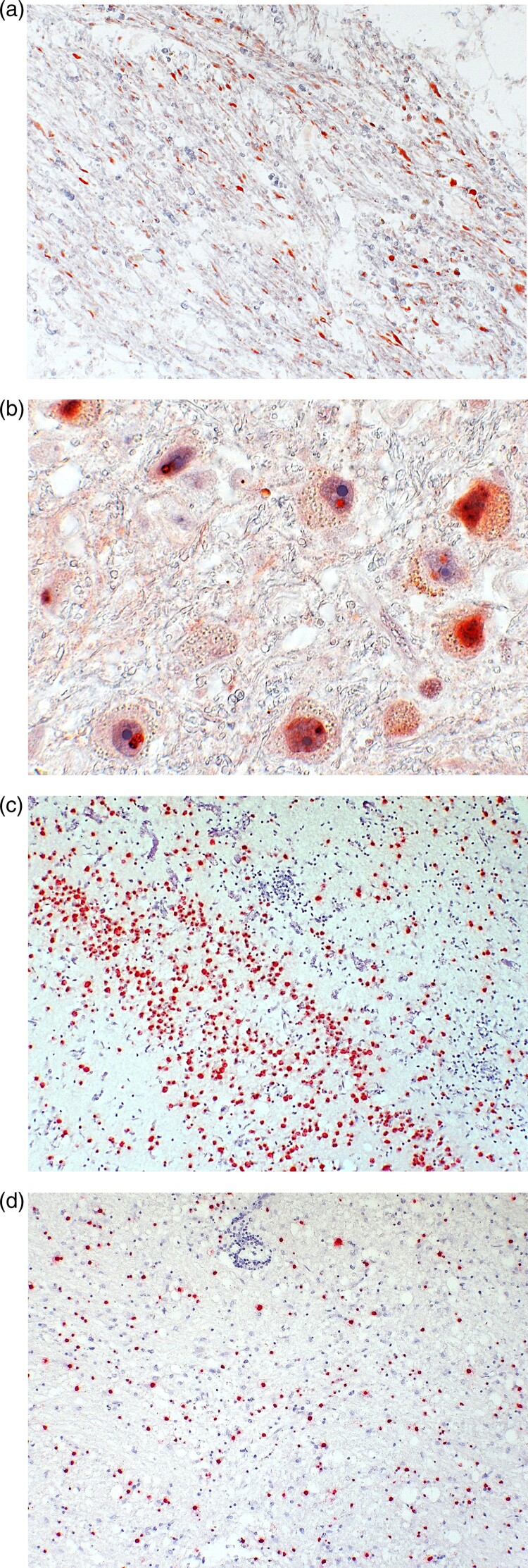
Detection of BoDV-1 antigen and RNA in brain tissue (case 2). A, Demonstration of viral antigen in neuropil and perikarya. Immunoperoxidase stain for BoDV-1 P antigen with light hematoxylin counterstain of the basal ganglia, original magnification x200. B, Presence of BoDV-1 antigen as spot-like nuclear inclusion or completely filling the nucleus. Immunoperoxidase stain for BoDV-1 P antigen with light hematoxylin counterstain of the pons, original magnification x1000. C, Detection of viral RNA. *In situ*-hybridization for BoDV-1 RNA of the hippocampus, original magnification x100. D, BoDV-1 RNA in the thalamus, *in situ*-hybridization, original magnification x100.

**Figure 6.**
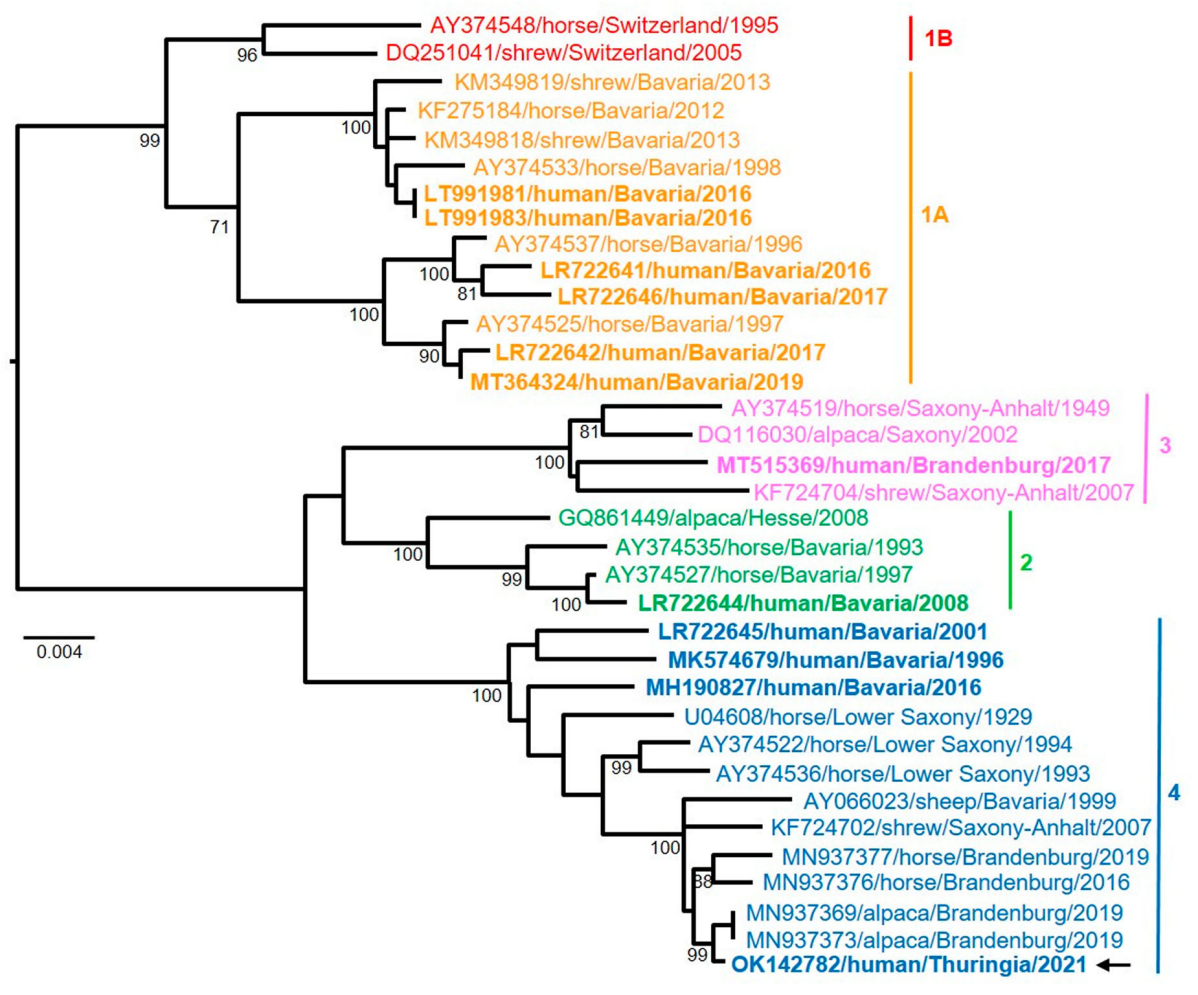
Maximum likelihood phylogenetic tree of BoDV-1 from Germany based on N and P gene nucleotide sequences (1824 nt, representing genome positions 54–1877 of BoDV-1 reference genome U04608). Cluster 1B (sequences from Switzerland) is shown in addition. Numbers at the nodes indicate maximum likelihood bootstrap replicates (>70%). GenBank accession number, host species, federal state and year of infection are provided per sequence. BoDV-1 sequences obtained from humans are shown in bold. The viral sequence of the case described herein is marked with an arrow, clustering with various animal sequences (cluster 4). Colour code of the clusters according to [6,7,11]. The scale bar indicates nucleotide substitutions per site.

**Case 2** was diagnosed in June 2021. The patient was a 79-year-old male pensioner from a rural region of Thuringia (Figure 1), who worked several hours per day in his allotment garden. He had not travelled outside Thuringia for 8 months. In early May 2021, he was hospitalized with malaise, headaches, cough, ataxia and progressive vigilance decline, soon followed by fever, epileptic seizures, loss of brainstem reflexes, and coma. Cranial MRI showed widespread signal intensity increases (Figure 3). IFAT titre was 2560 for serum and 640 for CSF. Line blot results for antibodies against BoDV-1 P were 34 AU for serum and 2 AU for CSF. A qRT-PCR analysis for BoDV-1 in CSF was positive (cycle quantitation (Cq) value of 32.4). The patient died 5 weeks after onset of illness, and diagnosis was made 6 days *post mortem*. Brain autopsy showed severe encephalitis (Figure 4A), characterized by perivascular and diffuse parenchymal T cell infiltration as well as mainly perivascular B cell accumulation (Figure 4B). T cell infiltrates consisted of CD4+ and CD8+ T cells (Figure 4C). Microglia activation was abundant (Figure 4D). Immunohistochemistry for BoDV-1 P antigen showed widespread positivity in the neuropil, perikarya and cell nuclei (Figure 5A,B) in all brain areas examined (frontal, parietal and occipital lobes, hippocampus, thalamus, basal ganglia, midbrain, pons, medulla, and cerebellum). *In situ*-hybridization demonstrated marked presence of viral RNA (Figure 5C,D) throughout the brain. A full BoDV-1 genome was recovered from FFPE tissue sections of the basal ganglia (GenBank accession no. OK142782) using NGS. Sequence and phylogenetic analysis placed this strain among BoDV-1 strains from shrews and domestic animals of cluster 4, which is endemic in Thuringia and the neighbouring federal states (Figure 6). According to the proposed case definition criteria [7], this case is a confirmed BoDV-1 encephalitis.

**Case 3** was notified in July 2021 and diagnosed retrospectively from a neuropathology archive. The patient was a 73-year-old woman residing in the extreme outskirts of a city in eastern Lower Saxony (Figure 1) for at least the last 30 years of her life. She fell ill and died in late 1992 of encephalitis with an unknown cause. Except for the presences of brain stem dysfunction no clinical information could be obtained, as the patient’s records had been destroyed more than a decade ago. Family members could not be identified for further information. Immunohistology for BoDV-1 P antigen was positive (not shown), as well as a qRT-PCR analysis (Cq value of 34.5). No full genome or sufficient partial genome for analysis could be recovered by NGS due to the advanced age of the sample. According to the proposed case definition criteria [7], case 3 is also a confirmed BoDV-1 encephalitis.

## Discussion

The epidemiology of human bornavirus encephalitis is still largely unknown. Based on a validated testing scheme [7] we here describe the detection of two acute human BoDV-1 encephalitis cases in BD endemic regions without previously recognized human infections. In addition, one case was retrospectively detected from archived brain tissue. The two acute cases were detected in 2021 during routine diagnostics of current encephalitis cases at the Bernhard Nocht Institute in Hamburg, following a nation-wide awareness campaign for clinicians, diagnostic laboratories and neuropathologists in 2019. The campaign used print media [15] and electronic information via e-mail for neurological and intensive care units. Prior to 2021, a prospective screening study from 2018 to 2020 for bornavirus infections in acute encephalitis cases of unknown etiology had detected three acute BoDV-1 encephalitis cases, all from the southern federal state of Bavaria [7]. All other previously published cases were also from Bavaria or with exposure linked to that specific federal state, except for one recently diagnosed case from a pathology archive in the federal state of Brandenburg in the north of Germany [11]. The now known four cases outside Bavaria are broadly distributed in the northern half of Germany’s established area endemic for animal BD. This clearly shows that human infection with BoDV-1 is not limited to Bavaria.

Despite the emerging character of BoDV-1 encephalitis – likely owing to increased awareness – human cases appear rare. A seroprevalence study in Bavaria showed a very low positive rate among veterinarians (0.14%) and no positivity in blood donors [16], possibly reflecting a high case-fatality ratio. How humans come into contact with the virus is unclear. Potential risk factors for BoDV-1 infection may be living in or close to rural areas, outdoor activities or agricultural work [6]. All three cases described in our report lived rurally, and case 2 was an avid gardener. The incubation period in humans is unknown, but ranges from a few weeks to months in naturally infected horses and alpacas [17,18]. Human BoDV-1 encephalitis cases are sporadic, and, except for the cluster of solid organ transplant-cases [4], remain without hints for a human-to-human transmission [6].

Recently, a serological testing scheme with updated graded case definitions for human bornavirus encephalitis has been published [7], based on different laboratory methods and consensus encephalitis criteria. Rapid *intra vitam* diagnosis of probable cases can be achieved by antibody detection in serum and CSF as illustrated here for case 1. The detection of bornavirus-reactive antibodies in serum is more sensitive than serology from CSF [7]. In addition, and mandatory for confirmed BoDV-1 infections, the direct detection of the virus by molecular techniques from CSF, as shown here in case 2, or brain tissue (biopsy or material obtained during autopsy) as described for cases 2 and 3, can be performed, as well as immunohistochemistry [7]. However, qRT-PCR from CSF is often negative [7], as demonstrated here in case 1, while positive from brain tissue [6,7]. The cranial MRI of patient 1 showed the typical features of BoDV-1 encephalitis [10], such as hyperintensities in the basal ganglia, thalamus and insula. Thus, a BoDV-1 infection of this patient is highly likely, though not yet molecularly confirmed. In a recent study with 19 BoDV-1 encephalitis patients from Bavaria, 53% showed MRI changes at a mean of 11 ± 10 days after symptom onset [10]. Thus, depending on the time the MRI is performed, typical changes may not be visible as exemplified by the case from Brandenburg [11].

Testing for BoDV-1 is often delayed due to lack of awareness, and the time to diagnosis may be several weeks while the patient’s disease progresses rapidly. In case 1 of our report, the time to diagnosis of three weeks was comparably short. In order to equip diagnostic laboratories with the skills and techniques to diagnose the infection, two external quality assurance tests have been successfully conducted for serologic and molecular testing [19,20]. BoDV-1 IFAT may be training-intensive in order to correctly recognize bornavirus-specific granular intranuclear fluorescence patterns. Specificity problems may arise due to anti-nuclear autoantibodies with a similar IFAT pattern, a problem which we circumvented by using an uninfected cell line in parallel [7,16], and well-characterized positive controls (serum and CSF from patients with proven BoDV-1 encephalitis). However, positive serology findings in apparently healthy people, not limited to the area of known animal BD [21–24] remain an unresolved issue. Antibodies against individual antigenic epitopes might be accidentally detected despite lack of any previous bornavirus contact [25]. In our study, we therefore examined only acutely ill patients with the clinical picture of encephalitis. We adhered to a specific case definition [7], thus increasing the positive pre-test probability, and combined two independent test systems, the IFAT and a line blot assay. Besides specificity, sensitivity of bornavirus serology is an issue. The time of seroconversion during human bornavirus encephalitis appears variable [4,6,7]. Patients may be seropositive at the time of hospitalization, or shortly before death of encephalitis. Thus, repeated testing is recommended in seronegative patients with a persisting suspicion of a BoDV-1 encephalitis [7]. Undeniably, serological tests being both highly sensitive and highly specific are needed. A variety of methods with differing sensitivity had been previously published in non-encephalitic patients, but none reached overall acceptance.

BoDV-1 genomes are highly conserved, with a high genetic similarity between different strains and isolates of ≥95%. Nonetheless, sequencing the BoDV-1 genome and subsequent phylogeographic analysis might provide clues about the region of infection [6,7,26]. Controversy exists on its applicability [27]. We here show that the BoDV-1 sequence of case 2 from Thuringia belongs to cluster 4, a cluster encompassing BoDV-1 sequences from infected shrews and domestic animals in the neighbouring federal states of Saxony-Anhalt and Lower Saxony, as well as from humans and domestic mammals from adjacent Bavaria and domestic mammals from Brandenburg [11]. In some federal states, different clusters are present. For case 3, no full or sufficient partial genome for analysis could be obtained by NGS.

Brain histology of case 2 showed the typical features of BoDV-1 encephalitis [9] which is morphologically indistinguishable from human encephalitis due to the related variegated squirrel bornavirus 1 (VSBV-1) [28]. In both encephalitis forms, marked inflammation with diffuse brain tissue infiltration and perivascular cuffing by T lymphocytes, combined with perivascular accumulation of B cells and strong microglial activation is present. While BoDV-1 is endemic in shrews in Germany, VSBV-1 was detected in captive exotic squirrel populations from Central America and Southeast Asia mainly in Germany. VSBV-1 has thus different reservoirs and a different epidemiology, fatally infecting private squirrel breeders and zoo animal caretakers who had contact to these animals [14,29–32]. Molecular tools and the line blot assay are able to discriminate between human BoDV-1 and VSBV-1 infections [7].

In conclusion, these three newly diagnosed human BoDV-1 encephalitis cases outside of Bavaria confirm that bornavirus infection has to be routinely considered as differential diagnosis in human encephalitis cases in all regions endemic for BD in livestock and areas known to harbour BoDV-1 positive shrews. Besides parts of northern and eastern Germany, this includes also virus-endemic regions in Austria, Liechtenstein, and Switzerland. It is suspected that most case-patients become infected close to their often markedly rural places of residence, but an incubation period of weeks to months may mean they could present with acute illness outside of BD endemic areas as well. BoDV-1 should be actively tested for in all acute encephalitis cases with residence or rural exposure history in known BD-endemic areas of Europe.
